# The Impact of Sodium-Glucose Co-Transporter-2 Inhibition on Insulin Resistance and Inflammation in Patients with Type 2 Diabetes: A Retrospective Study

**DOI:** 10.3390/medicina61020209

**Published:** 2025-01-24

**Authors:** Liana Iordan, Sandra Lazar, Romulus Timar, Simona Popescu, Teodora Sorescu, Oana Albai, Adina Braha, Bogdan Timar, Laura Gaita

**Affiliations:** 1 Doctoral School of Medicine, “Victor Babes” University of Medicine and Pharmacy, 300041 Timisoara, Romania; liana.iordan@umft.ro; 2 Second Department of Internal Medicine, “Victor Babes” University of Medicine and Pharmacy, 300041 Timisoara, Romania; timar.romulus@umft.ro (R.T.); popescu.simona@umft.ro (S.P.); sorescu.teodora@umft.ro (T.S.); albai.oana@umft.ro (O.A.); braha.adina@umft.ro (A.B.); bogdan.timar@umft.ro (B.T.); gaita.laura@umft.ro (L.G.); 3 Department of Diabetes, “Pius Brînzeu” Emergency County Hospital, 300723 Timisoara, Romania; 4 Centre for Molecular Research in Nephrology and Vascular Disease, “Victor Babes” University of Medicine and Pharmacy, 300041 Timisoara, Romania; 5 First Department of Internal Medicine, “Victor Babes” University of Medicine and Pharmacy, 300041 Timisoara, Romania; 6 Department of Hematology, Emergency Municipal Hospital, 300254 Timisoara, Romania

**Keywords:** insulin resistance, inflammation, sodium-glucose co-transporter 2 inhibitors, estimated glucose disposal rate

## Abstract

*Background and Objectives*: Insulin resistance (IR) is a key factor involved in the development of type 2 diabetes (T2D). Besides its role in the pathogenesis of T2D, insulin resistance is associated with impairment of glycemic control, reduced achievement of glycemic targets, and increases in cardiovascular risk and diabetes complications, being thus a negative prognosis factor. Sodium-glucose co-transporter-2 inhibitors (SGLT2i) are therapies for T2D which demonstrated, besides glycemic control, improvements of biomarkers traditionally associated with IR and inflammation. This study aimed to evaluate the impact of SGLT2i treatment on IR and inflammation biomarkers in patients with T2D. *Materials and Methods*: In a retrospective study, 246 patients with T2D treated with SGLT2i for a median of 5 years were evaluated regarding IR (estimated glucose disposal rate—eGDR, triglyceride/glucose index, triglyceride/HDLc index) and inflammation biomarkers (neutrophils to lymphocyte ratio, platelets to lymphocytes ratio and C-reactive protein) before and after intervention with SGLT2i. *Results*: After a median 5 years of SGLT2i treatment, patients with T2D had a higher eGDR (6.07 vs. 5.24 mg/kg/min; *p* < 0.001), lower triglyceride/HDLc ratio (3.34 vs. 3.52, *p* < 0.001) and lower triglyceride/glucose index (9.23 vs. 9.58; *p* < 0.001). The inflammation biomarkers decreased after SGLT2i therapy: C-reactive protein (3.07 mg/L vs. 4.37 mg/L), NLR (0.68 vs. 0.72; *p* < 0.001), and PLR (115 vs. 122; *p* < 0.001). Intervention with SGLT2i also improved the biomarkers associated with diabetes complications and cardiovascular risk: HbA1c (7.1% vs. 8.4%; *p* < 0.001), body mass index (30.0 vs. 31.5 kg/m^2^; *p* < 0.001) and urinary albumin to creatinine ratio (4.75 vs. 11.00 mg/g; *p* < 0.001). *Conclusions*: Treatment with SGLT2i in patients with T2D leads to decreases in IR and inflammation. These mechanisms may partially explain the additional cardiovascular and renal risk reductions associated with SGLT2i therapy, alongside the improvements in glycemic control, in patients with T2D.

## 1. Introduction

The 21st century has witnessed a shift in the main causes of mortality and morbidity worldwide, with increasing numbers of patients being affected by non-communicable diseases. One of these conditions, considered to be a pandemic of the modern world with estimates of 537 million patients in 2021 and a projection of 784 million patients in 2045, is diabetes, defined by chronic hyperglycemia and caused by an impaired pancreatic β-cell secretion or action of insulin or by a combination of the two, in a diversity of etiopathogenic, clinical and biological settings [1,2]. Diabetes contributes to a rise in the risk of cardiovascular disease (CVD) with an accelerated process of atherosclerosis and to chronic complications such as microangiopathy (diabetic retinopathy and diabetic kidney disease) and neuropathy, that are caused either directly through increased glycemic levels or indirectly through other risk factors that are frequently encountered especially in patients with type 2 diabetes (T2D) such as hypertension, dyslipidemia or obesity, leading to the necessity of a multifactorial therapeutic approach [2,3,4,5,6,7,8].

One of the antihyperglycemic classes of drugs is the sodium-glucose co-transporter-2 inhibitors (SGLT2i) that significantly inhibit the reabsorption of glucose and sodium in the proximal renal convoluted tubules, with consequences of a higher urinary excretion of glucose and a mild osmotic diuresis [9]. This insulin-independent mechanism of action, unique among other available antidiabetic agents, contributes to decreased glycemic values while avoiding hypoglycemia, decreasing body weight, lowering blood pressure, and, with paramount importance, as shown in various cardiovascular outcome trials (CVOTs), lowering the risk of major adverse cardiovascular events (MACE). The most common side effects include a mild increase in the risk of genital mycotic infections, euglycemic ketoacidosis, and, for canagliflozin, an increased risk of lower limb amputations [10,11,12,13]. However, it is considered that the SGLT2i have brought a revolution in the treatment of patients with T2D through their results in improving cardiorenal outcomes, results also shown in patients with chronic kidney disease (CKD) or heart failure (HF), regardless of the ejection fraction or the presence of diabetes. Moreover, these antihyperglycemic agents have proven beneficial effects in the majority of pathophysiological mechanisms that contribute to the development of T2D through the reduction in the tubular reabsorption of glucose itself, reduction in glucotoxicity, reduction in lipotoxicity, triglycerides and small-dense low-density lipoprotein cholesterol (LDLc), reduction in hepatic steatosis, metabolic reprogramming, reduction in plasma uric acid levels, hemodynamic effects (including modulation of the tubuloglomerular feedback) and direct renal and cardiac effects. [14,15,16,17,18,19,20,21,22,23,24,25,26].

One of the key benefits of SGLT2i is related to weight loss, especially since T2D is known to be strongly correlated with weight, with more than 80% of the patients presenting with either overweight or obesity, with increased adiposity and with an important visceral component [27,28,29]. With their contribution to an energy loss of approximately 280–360 kcal per day caused by the daily urinary loss of approximately 70–90 g of glucose, the SGLT2i can lead to a 1–5 kg weight reduction in a few months, with higher values being measured in patients with a higher hemoglobin A1c (HbA1c), although usually the weight loss observed in clinical practice is lower than the one expected from the quantity of glycosuria, and occasionally even temporary, effect partly explained by a potential increase in the subsequent energy intake [30]. Additionally, weight loss has also been encountered in patients without diabetes treated with SGLT2i, effects explained not only by the diuretic effect, but, in the absence of an important glycosuria, by multiple metabolic shifts such as the utilization of lipids for energy, that, as a consequence, will lead to mimicking a fasting state [31,32]. The weight loss consists mainly of fat mass—approximately 2/3 according to several studies—results that highlight the impact of this class of antidiabetic drugs in improving body composition in patients with T2D, alongside the beneficial results related to waist circumference (WC), body mass index (BMI), subcutaneous fat area and visceral fat area, with improved insulin sensitivity, lower values of fasting insulin, reduction in hepatic steatosis and quantitative and qualitative improvements of LDL and high-density lipoprotein (HDL) particles [10,14,33,34,35,36,37,38,39]. The beneficial effects of the SGLT2i have also been proven in patients with both genetic and acquired IR, since they are contributing to a decreased glucose toxicity, reduced oxidative stress, improvements in beta cell function and, as presented, to weight loss [40,41]. Moreover, another beneficial effect of SGLT2i is a direct and indirect decrease in inflammation, with lower levels of IL-1β, IL-6, monocyte chemoattractant protein-1 (MCP-1), matrix metalloproteinase-7, tumor necrosis factor-alpha (TNF-α), fibronectin-1, NFkB and profibrotic factors [42,43,44,45,46,47,48,49,50,51,52]. These anti-inflammatory effects of the SGLT2i have also been shown in animal models, where the treatment has led to reductions in the levels of C-reactive protein (CRP), alongside the other previously described markers, results also validated on human subjects [53,54].

Nevertheless, although these effects—and a plethora of others—have led to including SGLT2i as a first-line therapy in patients with T2D, CKD or HF, being considered disease-modifying drugs—most of the results are based on randomized controlled trials, with a lesser representation of Central-Eastern European countries such as Romania. With these premises, our study aimed to assess the impact of the treatment with SGLT2i in Romanian patients with T2D and to analyze its effects on cardiometabolic risk factors, with an emphasis on surrogate insulin resistance markers and inflammation.

## 2. Materials and Methods

### 2.1. Study Design and Patients

In this retrospective study, 246 patients with T2D treated with SGLT2i hospitalized in the Department of Diabetes, Nutrition and Metabolic Diseases of the “Pius Brînzeu” Emergency County Hospital in Timișoara, Romania, between July and September 2024, were enrolled consecutively. The design, protocol, and informed consent of this research have been previously approved by the hospital’s Ethics Committee (no. 471/8 July 2024). All of the included patients have provided written informed consent, while the study has been conducted according to the principles of the Declaration of Helsinki. The inclusion criteria were represented by an age > 18 years, a previous diagnosis of T2D, and an antihyperglycemic regimen that included an SGLT2i. In contrast, the exclusion criteria have been represented by the presence of another type of diabetes except T2D, the inability to provide informed consent, the inability to provide an accurate anamnestic medical history, the treatment with anti-inflammatory medication, such as nonsteroidal anti-inflammatory drugs, corticosteroids or immunosuppressants, and the absence of an SGLT2i from the treatment plan. The report of this study has been prepared according to the STROBE (Strengthening the reporting of observational studies in epidemiology) statement checklist [55].

### 2.2. Clinical, Anthropometric, and Laboratory Data

The data regarding patients’ sex, age, duration of T2D, weight, and waist circumference (WC) have been collected from their medical records. The body mass index (BMI) was calculated as weight (kg)/height^2^ (m). Hypertension was defined as systolic blood pressure ≥ 140 mmHg and/or diastolic blood pressure ≥ 90 mmHg, a self-reported history of physician-diagnosed hypertension and/or the use of antihypertensive agents, while the systolic blood pressure (SBP) and diastolic blood pressure (DBP) measurements were performed according to the recommendations of the European Society of Cardiology [56]. Moreover, the estimated glomerular filtration rate (eGFR) was calculated using the CKD-EPI Creatinine Equation (2021), as follows: eGFR = 142 × min(standardized Scr/K,1)^α^ × max(standardized Scr/K,1)^−1.200^ × 0.9938^age in years^ × 1.012 [if female], where Scr = serum creatinine in mg/dL, K = 0.7 (females) or 0.9 (males), α = −0.241 (females) or −0.302 (males), min(standardized Scr/K,1) = the minimum of Scr/K or 1 and max(standardized Scr/K,1) = the maximum of Scr/K or 1 [57].

Additionally, the fasting plasma glucose (FPG), HbA1c, LDLc, HDLc, triglycerides, CRP, and complete blood count (CBC) have been measured using standardized methods in the “Pius Brînzeu” Emergency County Hospital laboratory, after at least 12 h of fasting, while the urinary albumin/creatinine ratio was measured from a spot urine sample, using standardized methods in the same laboratory.

### 2.3. Insulin Resistance and Inflammation Assessment

Although the accepted standard for measuring insulin resistance is the euglycemic-hyperinsulinemic clamp, this procedure is not feasible for clinical practice or epidemiological studies. At the same time, the Homeostasis Model Assessment of Insulin Resistance (HOMA-IR) requires the measurement of serum insulin, which is not routinely performed in Romanian healthcare facilities. Nevertheless, various simple, cost-effective surrogate markers have been validated, some of them showing a better correlation with the euglycemic-hyperinsulinemic clamp than the HOMA-IR, such as the triglyceride/high-density lipoprotein cholesterol (TG/HDL-C) ratio or the triglyceride-glucose index (TyG). In this study, the TyG index was calculated according to the formula: ln [fasting triglycerides (mg/dL) × fasting plasma glucose (mg/dL)/2] [58].

Moreover, the estimated glucose disposal rate (eGDR), another surrogate marker for insulin resistance was calculated in our study with the formula: eGDR (mg/kg/min) = 21.158 − (0.09 × WC) − (3.407 × HT) − (0.551 × HbA1c), where WC = waist circumference (cm), HT = hypertension (yes = 1/no = 0), and HbA1c = hemoglobin A1c (%). This marker, which has demonstrated a strong correlation with the hyperinsulinemic-euglycemic clamp, was firstly developed in patients with T1D and then tested in patients with T2D [59,60,61,62].

Regarding the assessment of inflammation, alongside the measurement of CRP, neutrophil-to-lymphocyte ratio (NLR—the ratio of neutrophil count to lymphocyte count) and platelet-to-lymphocyte ratio (PLR—the ratio of platelet count to lymphocyte count) have been calculated in this study, as additional inflammation biomarkers [63].

### 2.4. Statistical Analysis

For the statistical analysis, the MedCalc^®^ Statistical Software version 20.210 has been used (MedCalc Software Ltd., Ostend, Belgium; https://www.medcalc.org (accessed on the 22 September 2024); 2022). The numerical variables with Gaussian distribution are represented as mean ± standard deviation, while the numerical variables with non-parametric distribution are median and [interquartile range]. In order to assess the normality of the distribution of numerical variables, we used the Shapiro and Wilk method, with a *p*-value lower than 0.05, which corresponded to a non-parametric distribution.

In order to evaluate the significance of differences between the two groups, the Mann–Whitney U test was used to compare two medians in non-parametric populations and the Student *t*-test to compare arithmetic means in Gaussian populations. In contrast, the paired samples Wilcoxon test (or Wilcoxon signed-rank test) was used to evaluate the significance of the median difference between the two paired samples.

To obtain a confidence level of 95% and a statistical power higher than 80% the sample size was calculated before the enrollment, taking into account the information found in the literature [64]. In this study, the *p*-value threshold under which statistical significance was considered is 0.05.

## 3. Results

The enrolled patients’ characteristics before initiating SGLT2i therapy are presented in Table 1. Men had a significantly higher weight (100 vs. 80 kg; *p* < 0.001; Mann–Whitney U test), BMI (32 vs. 31 kg/m^2^; *p* < 0.001), waist circumference (100.8 vs. 85.9 cm; *p* < 0.001) and a lower LDLc (79.5 vs. 84.0 mg/dL; *p* = 0.032). No other significant differences between men and women were observed in the studied group.

No significant differences regarding inflammation markers between men and women were observed before initiating SGLT2i therapy in the studied group (Table 2).

Before starting the treatment with SGLT2i, men had a significantly lower eGDR (4.30 vs. 5.86 mg/kg/min; *p* < 0.001) and significantly higher TyG index (9.63 vs. 9.56; *p* = 0.02), while the TG/HDLc index was marginally, although not significantly, higher (*p* = 0.068) in men (3.61) vs. women (3.45). The comparison between IR biomarkers in patients with T2D before starting SGLT2i is presented in Table 3.

**Figure 1:**
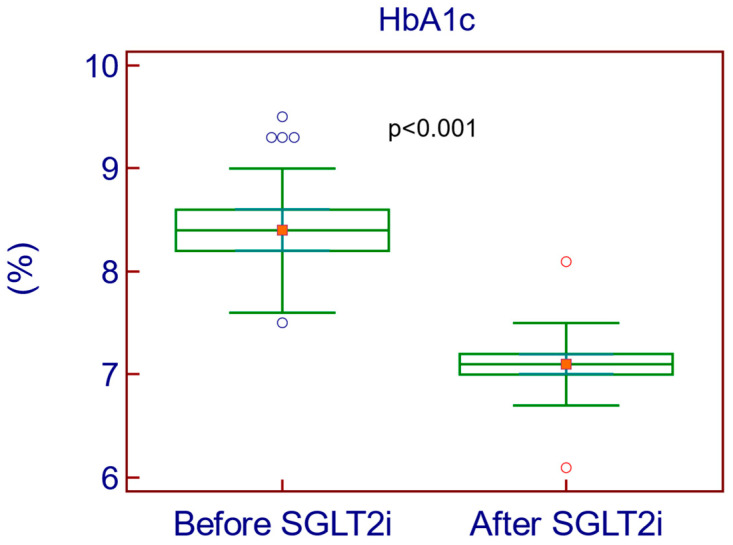
Decrease in HbA1c after SGLT2i treatment.

The treatment with SGLT2, for a median duration of 5 years, with an interquartile range of 4 to 6 years, resulted in significant improvements in risk biomarkers associated with diabetes complications and CV events (weight, BMI, WC, SBP, DBP, FPG, HbA1c (Figure 1), LDLc, triglycerides, and UACR). The detailed comparison of these biomarkers before and after SGLT2i treatment is presented in Table 4 and Table 5.

**Figure 2:**
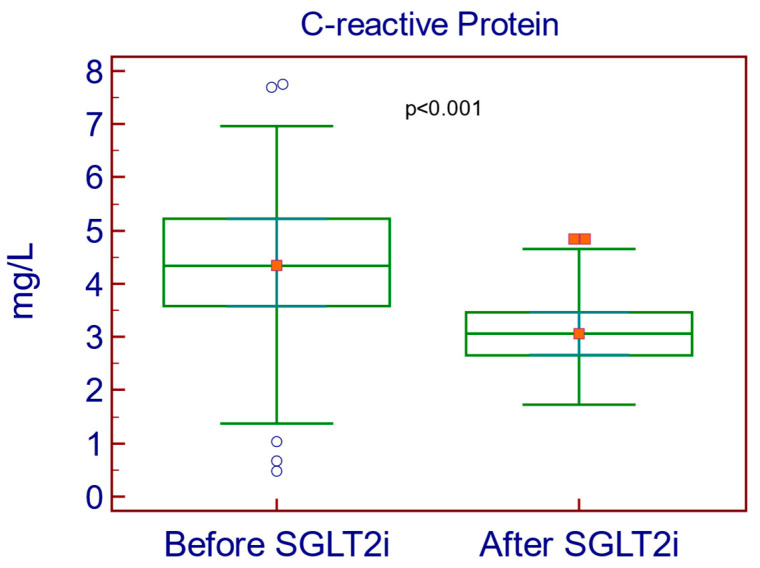
Decrease in C-reactive protein after treatment with SGLT2i.

SGLT2i intervention in patients with T2D led to improvements in inflammation biomarkers by decreasing the NLR (0.68 vs. 0.72; *p* < 0.001), PLR (115 vs. 122; *p* < 0.001) and C-reactive protein (3.07 mg/L vs. 4.37 mg/L; *p* < 0.001: Figure 2).

**Figure 3:**
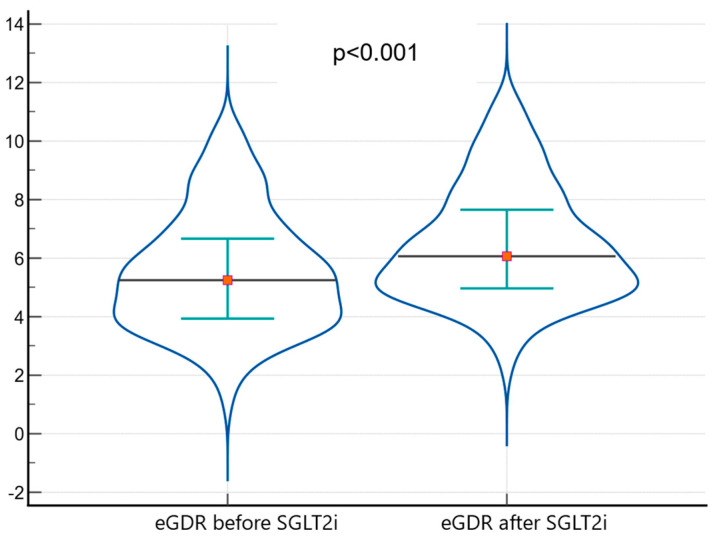
Increases in eGDR after SGLT2i therapy.

After the treatment with SGLT2i, patients with diabetes had a significantly decreased IR as described by a significantly higher eGDR (6.07 vs. 5.24 mg/kg/min; *p* < 0.001; Figure 3), respectively, significantly lower TG/HDLc ratio (3.34 vs. 3.52, *p* < 0.001) and TyG index (9.23 vs. 9.58; *p* < 0.001).

## 4. Discussion

### 4.1. Findings and Their Interpretation

Results of this study demonstrated that women had significantly higher eGDR compared to men, indicating that women exhibit better insulin sensitivity. This finding is clinically significant, as IR is a central feature of T2D and a key target for therapeutic interventions.

Despite not being a medication that directly targets the phenomenon of IR, SGLT2i has been observed to have a beneficial impact on the biomarkers associated with IR, such as weight, BMI, TG/HDL ratio, and eGDR, among individuals with T2D [36,65]. These findings, also identified by this present study, have been mentioned in various articles which focus on the benefits of SGLT2i on weight reduction, especially the reduction in visceral adipose tissue, the improvement of hepatic steatosis and metabolic shifts such as the increase in lipolysis and β-oxidation of fatty acids, with a consecutive increase in ketone bodies, with some studies reporting favorable effects on the skeletal muscle insulin sensitivity [66,67,68,69,70]. Furthermore, these medications have improved various indicators frequently linked to inflammatory parameters, such as CRP, NLR, and PLR, not only in this study, but also in multiple other trials performed on animal models and human subjects [71,72,73,74]. This underscores the important role of SGLT2i in managing T2D, as their medium and long-term effects extend beyond the direct impact on glycemic levels [75]. These findings suggest that the beneficial effects of SGLT2i can be mediated through the improvement of IR and the attenuation of the associated inflammatory processes that often occur in individuals with IR.

Moreover, some of these biomarkers associated with IR have also been linked to the progression of HF and CKD [76,77]. Since therapy with SGLT2i has shown a direct beneficial impact on reducing the risk and progression of these cardiovascular and renal impairments, even in individuals without DM, the outcomes of this study suggest that the beneficial effects of SGLT2i on HF and CKD may be partially explained by their ability to improve IR, in addition to the already known mechanisms of action [78].

TG/HDL ratio is a well-established biomarker that highly impacts IR [79]. The study findings demonstrated a statistically significant inverse relationship between TG/HDL ratio and insulin sensitivity, indicating that a more atherogenic lipid profile is associated with decreased insulin sensitivity. Moreover, since this report is a simple, cost-effective, and easily accessible parameter, it could serve as a screening tool to select patients who may benefit from more detailed assessments of IR, such as through the use of HOMA-IR or eGDR, which are more complex and resource-intensive to perform, highlighting the potential clinical utility of the TG/HDL ratio in identifying and monitoring IR among individuals with T2D, which could inform targeted management strategies and improve patient outcomes [80].

### 4.2. Strengths and Weaknesses

This study provides a comprehensive analysis of the impact of iSGLT2 on various biomarkers associated with IR in individuals with T2D. It provides novel insights by exploring the effect of iSGLT2 therapy on less commonly studied parameters, such as CRP, NLR, PLR, or TG/HDL ratio, all of which are closely linked to inflammation, IR, and cardiometabolic outcomes. Furthermore, the study focuses on the examination of these effects on a specific population, namely individuals with T2D from (Central Eastern Europe) Romania, that has not been extensively investigated from this perspective, providing valuable insights into the broader clinical implications of SGLT2i therapy.

The weaknesses of this study are represented by the fact that this study has a retrospective design, which may limit the ability to infer causal relationships between SGLT2i use and the observed improvements in IR biomarkers. Additionally, the duration of exposure to SGLT2i was not standardized across all patients, which could introduce variability and potentially confound the findings. However, this study has a median exposure duration of 5 years, with an interquartile range of 4 to 6 years, indicating a degree of homogeneity in the sample. Another weakness could be represented by the use of surrogate IR markers such as the eGDR, which was firstly developed in patients with T1D and then tested, yet still insufficiently, in patients with T2D. In this context, the validation of the eGDR in different populations of patients with T2D will represent the starting point of future research. Lastly, this study did not include data regarding the additional antihyperglycemic agents of the subjects; nevertheless, these aspects are going to be considered in future studies, since the impact of certain antidiabetic drugs such as metformin or glucagon-like peptide-1 receptor agonists on IR and inflammation is well-known.

### 4.3. Relevance of the Findings: Implications for Clinicians and Policymakers

The observed sex differences in insulin sensitivity may be attributed to various physiological and hormonal factors. For instance, the current literature data indicate that pre-menopausal women tend to have increased insulin sensitivity compared to men, likely due to the protective effects of female sex hormones such as estrogen. Estrogen has been shown to enhance insulin signaling and glucose uptake in peripheral tissues. However, this protective effect diminishes during the menopausal transition, contributing to the increased IR often seen in post-menopausal women [81,82,83].

In addition to hormonal influences, differences in lifestyle factors between men and women may also play an important role in the observed variations in IR. Differences in body composition, fat distribution, physical activity levels (men tend to be more sedentary than women), dietary intake (women typically exhibit healthier dietary habits compared to men), and other lifestyle behaviors (including exposure to substances such as alcohol and tobacco) between the sexes can all impact insulin sensitivity. For example, men tend to have greater muscle mass and central fat accumulation, which are associated with higher IR. In contrast, women typically have more peripheral fat distribution, which is less detrimental to insulin action. Nevertheless, these sex-specific physiological, hormonal, and lifestyle factors highlight the importance of their consideration when evaluating and managing IR in individuals with T2D [81,84,85].

Moreover, the intensively studied relationship between IR and inflammation reveals a stronger association [86]. Even though the precise pathophysiological mechanisms underlying the interconnection between the initiation of inflammation and IR are not fully elucidated, existing research has demonstrated that individuals with IR exhibit elevated levels of various pro-inflammatory cytokines, along with a concomitant decrease in anti-inflammatory cytokines [87]. This pro-inflammatory state appears to be a key contributor to the development and progression of IR. Similarly to this study’s findings, non-specific inflammatory biomarkers such as CRP, NLR, and PLR are significantly elevated in the context of IR [88].

On the other hand, thrombocytes are activated in chronic inflammatory processes due to various stimuli, which can increase the risk of thrombosis formation in both micro- and macrovascular territories [89,90,91]. This is particularly concerning, as IR and T2D are known to play a significant role in developing microvascular and macrovascular comorbidities, such as CKD and cardiovascular disease [92]. The increased inflammation linked to IR, coupled with the heightened thrombotic activity, creates a self-perpetuating cycle that can ultimately lead to the development of serious comorbidities [93,94]. This observation highlights the potential value of these easily accessible inflammatory biomarkers as proxy measures of IR, which could enhance their clinical application for individuals with T2D.

Regarding treatment strategies in T2D, this study sheds light on additional benefits of SGLT2i beyond their well-established effects on DM complications, cardiovascular outcomes, and renal impairment, indicating that SGLT2i can also improve markers of IR and inflammation, which are prior pathophysiological mechanisms underlying T2D and its associated comorbidities [95,96]. By elucidating these effects, the study provides a more comprehensive understanding of how SGLT2i can optimize metabolic control and reduce the risk of adverse outcomes in individuals with T2D.

Moreover, the study identifies a set of easily measured indicators that can serve as reliable biomarkers of IR and provide practical screening tools for identifying individuals with increased IR who may require more targeted interventions or closer monitoring.

## 5. Conclusions

The treatment with SGLT2i reduces the IR and inflammation in patients with T2D. These mechanisms may partially explain the previously demonstrated beneficial effects of SGLT2i on heart failure and cardiovascular and renal risk, alongside the improvements in glycemic control, in patients with T2D.

## Figures and Tables

**Table 1:** Patients’ characteristics before initiating SGLT2i therapy.

Variable	Entire Group (n = 246)	Men (n = 134)	Women (n = 112)	*p* (Men vs. Women)
Age (years) ^a^	63.6 ± 8.3	62.6 ± 8.2	64.8 ± 8.3	0.043
Diabetes duration (years) ^b^	8 [5; 14]	8 (119.5)	9 (128.3)	0.329
Weight (kg) ^b^	91 [80; 102]	100 (160.7)	80 (79)	<0.001
BMI (kg/m^2^) ^b^	31.5 [31; 32.5]	32.0 (139.2)	31.0 (104.7)	<0.001
Waist circumference (cm) ^a^	94.0 ± 13.6	100.8 ± 10.7	85.9 ± 12.3	<0.001
SBP (mmHg) ^b^	140.0 [134–143]	139.5 (120.8)	140.0 (126.7)	0.514
DBP (mmHg) ^b^	75 [70–85]	75 (119.9)	75 (127.7)	0.387
FPG (mg/dL) ^b^	179 [162; 193]	180 (129)	178 (116.9)	0.183
HbA1c (%) ^b^	8.4 [8.2; 8.6]	8.4 (124.4)	8.4 (122.4)	0.829
LDLc (mg/dL) ^b^	81.0 [76; 89]	79.5 (114.6)	84.0 (134.1)	0.032
Tg (mg/dL) ^a^	168.4 ± 32	170.9 ± 31.5	165.4 ± 32.5	0.179
HDLc (mg/dL) ^b^	47 [43; 53]	46 (119.4)	47 (128.4)	0.323
eGFR (mL/min/1.73 m^2^) ^b^	79.0 [70; 90]	78.5 (118.4)	80.5 (129.5)	0.224
UACR (mg/g) ^b^	11.00 [6.7; 22]	11.00 (123.7)	11.20 (123.3)	0.967

^a^ Continuous variable with Gaussian distribution. Results are presented as mean ± standard deviation. The *p*-value was calculated using the unpaired *t*-student’s test. ^b^ Continuous variables with non-parametric distribution. Results are presented as median, [interquartile-range] and (average rank). The *p*-value was calculated using the Mann–Whitney U test. BMI: body mass index; SBP: systolic blood pressure; DBP: diastolic blood pressure; FPG: fasting plasma glucose; HbA1c: hemoglobin A1c; LDLc: low-density lipoprotein cholesterol; Tg: triglycerides; HDLc: high-density lipoprotein cholesterol; eGFR: estimated glomerular filtration rate; UACR: urinary albumin/creatinine ratio.

**Table 2:** Inflammation markers before initiating SGLT2i therapy.

Variable	Entire Group	Men	Women	*p* (Men vs. Women)
CRP (mg/L) ^a^	4.37 ± 1.28	4.30 ± 1.3	4.40 ± 1.2	0.329
NLR ^b^	0.72 [0.64; 0.74]	0.72 (121.1)	0.70 (126.3)	0.563
PLR ^b^	122 [115; 125]	122 (122.4)	122 (124.8)	0.788

^a^ Continuous variable with Gaussian distribution. Results are presented as mean ± standard deviation. The *p*-value was calculated using the unpaired *t*-student’s test. ^b^ Continuous variables with non-parametric distribution. Results are presented as median, [interquartile range], and (average rank). The *p*-value was calculated using the Mann–Whitney U test. CRP: C-reactive protein; NLR: neutrophil-to-lymphocyte ratio; PLR: platelet-to-lymphocyte ratio.

**Table 3:** Biomarkers of insulin resistance before initiating SGLT2i therapy.

Variable	Entire Group	Men	Women	*p* (Men vs. Women)
TG/HDLc ^a^	3.52 [2.94; 4.24]	3.61 (131.1)	3.45 (114.5)	0.068
TyG index ^b^	9.58 ± 0.23	9.63 ± 0.22	9.56 ± 0.23	0.020
eGDR (mg/kg/min) ^a^	5.24 [3.94; 7.17]	4.30 (100.9)	5.86 (150.5)	<0.001

^a^ Continuous variable with the non-parametric distribution. Results are presented as median, [interquartile range] and (average rank). The *p*-value was calculated using the Mann–Whitney U test. ^b^ Continuous variable with Gaussian distribution. Results are presented as mean ± standard deviation. The *p*-value was calculated using the unpaired *t*-student’s test. TG/HDLc: triglyceride/high-density lipoprotein cholesterol; TyG index: triglyceride glucose index; eGDR: estimated glucose disposal rate.

**Table 4:** Impact of SGLT2i on diabetes and cardiovascular risk and insulin resistance biomarkers.

	Before SGLT2i	After SGLT2i	Paired Difference	
			Difference (% Difference)	*p*-Value
Weight (kg)	91	89	−2 kg (2.22%)	<0.001
BMI (kg/m^2^)	31.5	30.0	−1.5 kg/m^2^ (4.88%)	<0.001
SBP (mmHg)	140.0	135.0	−5.0 mmHg (3.64%)	<0.001
DBP (mmHg)	75	70	−5 mmHg (6.90%)	<0.001
FPG (mg/dL)	179	128	−51 mg/dL (33.22%)	<0.001
HbA1c (%)	8.4	7.1	−1.3% (16.77%)	<0.001
HDLc (mg/dL)	47	49	+2 mg/dL (4.17%)	<0.001
eGFR (ml/min/1.73 m^2^)	79.0	73.0	−6.0 mL/min (7.89%)	<0.001
UACR (mg/g)	11.00	4.75	−6.25 mg/g (79.37%)	<0.001
NLR	0.72	0.68	−0.04 (5.71%)	<0.001
PLR	122	115	−7 (5.91%)	<0.001
TyG index	9.58	9.23	−0.35 (3.72%)	<0.001
eGDR (mg/kg/min)	5.24	6.07	+0.83 (14.68%)	<0.001

The *p*-value was calculated using the paired Wilcoxon test. BMI: body mass index; SBP: systolic blood pressure; DBP: diastolic blood pressure; FPG: fasting plasma glucose; HbA1c: hemoglobin A1c; HDLc: high-density lipoprotein cholesterol; eGFR: estimated glomerular filtration rate; UACR: urinary albumin/creatinine ratio; NLR: neutrophil-to-lymphocyte ratio; PLR: platelet-to-lymphocyte ratio; TyG index: triglyceride glucose index; eGDR: estimated glucose disposal rate.

**Table 5:** Impact of SGLT2i therapy on inflammation and cardiovascular risk biomarkers.

	Before SGLT2i		After SGLT2i		Paired Difference			
	Mean	SD	Mean	SD	Mean	SD	95% CI	*p*-Value
Waist circumference (cm)	94.0	13.6	91.2	13	−2.8	1.9	−3.09 to −2.61	<0.001
Triglycerides (mg/dL)	168.4	32	159.8	31.6	−8.6	18.9	−11.05 to −6.31	<0.001
C-reactive protein (mg/L)	4.37	1.27	3.07	0.62	−1.3	1.41	−1.47 to −1.12	<0.001

Continuous variable with Gaussian distribution. *p*-value was calculated using the paired sample *t*-student’s test.

## Data Availability

The data presented in this study are available on request from the corresponding author. The data are not publicly available due to local privacy and data protection regulations.
